# Characterization of Chicken Skin Yellowness and Exploration of Genes Involved in Skin Yellowness Deposition in Chicken

**DOI:** 10.3389/fphys.2021.585089

**Published:** 2021-03-31

**Authors:** Jingwen Wu, Zetong Lin, Genghua Chen, Qingbin Luo, Qinghua Nie, Xiquan Zhang, Wen Luo

**Affiliations:** ^1^Department of Animal Genetics, Breeding and Reproduction, College of Animal Science, South China Agricultural University, Guangzhou, China; ^2^Guangdong Provincial Key Lab of Agro-Animal Genomics and Molecular Breeding, and Key Lab of Chicken Genetics, Breeding and Reproduction, Ministry of Agriculture and Rural Affairs, South China Agricultural University, Guangzhou, China

**Keywords:** chicken, skin color, yellowness value, pigmentation, candidate gene

## Abstract

Skin color is an important economic trait in meat-type chickens. A uniform bright skin color can increase the sales value of chicken. Chickens with bright yellow skin are more popular in China, especially in the broiler market of South China. However, the skin color of chickens can vary because of differences in breeds, diet, health, and individual genetics. To obtain greater insight into the genetic factors associated with the process of skin pigmentation in chickens, we used a colorimeter and high-resolution skin photographs to measure and analyze the skin color of chickens. By analyzing 534 chickens of the same breed, age, and feed condition, we found that the yellowness values of the chickens varied within this population. A significant positive correlation was found between the cloacal skin yellowness values before and after slaughter, and the cloacal skin yellowness value of live chickens was positively correlated with the overall body skin yellowness value. Additionally, chicken skin yellowness exhibited low heritability, ranging from 0.07 to 0.27. Through RNA sequencing, 882 genes were found to be differentially expressed between the skin with the highest and lowest yellowness values. Some of these differentially expressed genes may play an important role in yellow pigment deposition in chicken skin, which included *TLR2B, IYD, SMOC1, ALDH1A3, CYP11A1, FHL2, TECRL, ACACB, TYR, PMEL*, and *GPR143*. In addition, we found that the expression and variations of the *BCO2* gene, which is referred to as the yellow skin gene, cannot be used to estimate the skin yellowness value of chickens in this population. These data will help to further our understanding of chicken skin yellowness and might contribute to the selection of specific chicken strains with consistent skin coloration.

## Introduction

Ma-Huang chicken is a high-quality broiler chicken with golden skin that is preferred by consumers and is one of the most popular chickens in the frozen chicken market of South China. Under the condition of an increasing frozen fresh chicken market, the first impression that a broiler presents to consumers is made by its skin. The yellowness and uniformity of skin color are two important factors influencing consumer choice. Many factors can regulate skin color, and skin yellowness value varies greatly between individuals. The market price of chickens with white or light yellow skin is lower than that of chickens with yellow skin. How to select for uniform skin color and generate chickens with higher yellowness is a critical problem limiting the development of commercial Ma-Huang chickens.

Coloration is a phenotypic trait related to a variety of adaptive functions, including temperature regulation, camouflage, mate choice, etc (Hill, 1991; Hamilton et al., 2013). Skin pigmentation is a complex biological process. Skin color can be influenced by environmental factors, nutrition, and heredity. Carotenoids are compounds responsible for skin color in animals. Most animals can only acquire carotenoid pigments from the environment. Carotenoids can be synthesized by plants, bacteria, and fungi and affect the red, orange, and yellow characteristics of the skin (Goodwin, 1954; Walsh et al., 2012; Toews et al., 2017). Chickens obtain carotenoids through eating feeds. Once ingested, these pigments are transported to the mucosal cells of the duodenum. Scavenger receptor class B, type 1 can recognize carotenoids incorporate with lipid micelles and facilitate carotenoids transportation (Eroglu and Harrison, 2013; Toomey et al., 2017). Once absorbed, carotenoids are transported by lipoproteins through the bloodstream to the target tissues. The abundance and nature of lipoproteins can limit the capacity to obtain carotenoids during this process (Galvan et al., 2016). Furthermore, many species can metabolize carotenoids into alternate, more oxidized forms that are deposited into the integument. The result is that several factors, such as amounts and species of carotenoids ingested, nutrient and health status of the host, genetic background, and food matrix composition, influence the bioavailability of carotenoids (West and Castenmiller, 1998). Therefore, there are large differences between species or individuals in their capacity to obtain these pigments from their diets, resulting in skin color variation. However, unlike endogenously synthesized pigments such as melanin, which have well-characterized genotype-phenotype connections, researchers were poorly understood the genes and pathways involved in exogenously derived carotenoid metabolism and deposition (Toews et al., 2017).

β-Carotene oxygenases are the best-characterized set of enzymes that are involved in carotenoid metabolism, including β-carotene 15, 15′ oxygenase (BCO1) and β-carotene 9′,10′ oxygenase (BCO2) (Toews et al., 2017). *BCO2* is a mitochondrial protein with a set of varied targets, including zeaxanthin, lutein, and lycopene. *BCO2* is able to directly influence coloration by asymmetrically decomposing colored carotenoids into colorless defatted carotenoids, which hinder the deposition of colored pigments in fat and skin (Amengual et al., 2011). In domesticated chickens, lower expression of *BCO2* is associated with carotenoid degradation into colorless derivatives (Eriksson et al., 2008). Eriksson et al. (2008) analyzed gene sequences associated with a specific phenotype found in domestic chickens across numerous wild junglefowl and domestic breeds and found that three SNPs in *BCO2* were significantly related to yellow skin [located at chr24: 6,264,085 (G < A), chr24: 6,273,428 (A < G), and chr24: 6.287.900 (G < A)]. The presence of *BCO2* initiates the degradation of carotenoids and protects mitochondria from oxidative damage (Harrison and Kopec, 2020). Although the above studies have indicated the importance of *BCO2* in chicken yellow skin regulation, its function in the regulation of skin pigmentation and skin yellowness value still remains unclear.

In the present study, we systematically analyzed the variation in skin yellowness in yellow-skinned broiler chickens under intensive conditions. We found that the yellowness values of skin varied within the chicken population under intensive conditions. To identify the candidate genes that influence yellowness in chicken skin, we used RNA-seq to investigate differences in skin transcriptomes between the skin tissues of the Ma-Huang chicken showing high yellowness and low yellowness. We identified some candidate genes and metabolic pathways involved in yellow pigment deposition in chickens. All of these findings will help to establish a foundation for breeding chickens with high yellow skin coloration.

## Materials and Methods

### Ethics Statement

Ma-Huang chickens were obtained from a chicken farm in Guangzhou, Guangdong Province, China. Animal experiments were performed in accordance with the regulations and guidelines established by the Animal Care Committee of South China Agricultural University (approval number: SCAU#0106; 25 November 2018).

### Animals and Traits Measurement

Commercial Ma-Huang chicken pure line broiler chickens were used in the present study. Chickens at 120 days of age raised under the same feeding management conditions were chosen. Slaughter tests and traits measurements were carried out in a total of 534 Ma-Huang chickens (24 males and 510 females). These chickens were from one generation with complete pedigree. All chickens were slaughtered by bloodletting in the jugular vein, then scalded in tanks filled with water at a temperature of 64°C for 220 s and plucked with a chicken defeatherer. The slaughter determination was analyzed using methods adapted from previous studies (Sirri et al., 2010). Growth traits and carcass traits were measured according to the NY/T 823-2004 standard (Chen et al., 2004).

The skin yellowness was measured using a 3nh-NH310 colorimeter (3NH, Guangzhou, China). The colorimeter can directly obtain a^∗^ value (which represents redness), b^∗^ value (which represents yellowness), and L^∗^ value (which represents luminance) from a single measurement. We measure the skin yellowness by b^∗^ value. To reduce measurement errors, skin color values were determined by the same person, and quantification was performed three times at each position. The skin b^∗^ values (yellowness) of the cloaca (for living chickens) was measured before bloodletting. This area was chosen because it contains a few feathers or blood vessels. After bloodletting, scalding and plucking, the skin b^∗^ values (yellowness) of eight different parts were measured. All color measurement areas were free from obvious defects (plucking damage, bruises, full blood vessels, hemorrhage, discoloration, and any other condition that might affect a uniform color reading).

### Digital Skin Color Value Quantification

Digital photographs of 349 slaughtered chickens, placed on a white disk as the background, were successfully obtained with a Canon EOS 80D color camera (Canon, Tokyo, Japan). We developed an in-house program in MATLAB 2018a (The MathWorks, Inc., Natick, MA, United States) to automatically retrieve the color values of the chicken skin. RGB values were converted into HSV space according to standard formulas implemented by the rgb2hsv function of MATLAB (Supplementary Figure 1). Median HSV values, H values, S values, and V values were then reported for each image. In the HSV model, H represents hue (H represents the variation of the color type), S is saturation (S represents the purity or intensity of the color), and V is brightness or luminance. The V values were observed under environmental light, which could not be fully controlled, so we focused on the H and S color dimensions. A photograph is provided in Supplementary Figure 2 as an example. The MATLAB binary “get_skin_color” is provided in the Supplementary Material.

### DNA and RNA Extraction

Genomic DNA was extracted using the DNeasy Blood & Tissue Kit (Qiagen, CA, United States). Total RNA was extracted from tissues using RNAiso reagent (Takara, Otsu, Japan) and quantified using a NanoDrop spectrophotometer and an Agilent 2100 Bioanalyzer (Thermo Fisher Scientific, MA, United States).

### Genetic Parameter Estimates

The restricted maximum likelihood method implemented by the DMU package was used to obtain estimates of the phenotypic and genetic (co)variance and heritability (Madsen and Jensen, 2013), and the following linear model was used for the analysis of the data:

y=Xb+Za+e

where y was the phenotypic value of a trait, a was the vector of the animal additive genetic effect, b was the vector of the fixed effects, including gender (two levels) and pen (six levels), e was the vector of random residuals, and X and Z were the incidence matrices. A 534 individuals with complete pedigree and phenotypic trait records were included.

### RNA-seq and Bioinformatics Analysis

Among the 534 Ma-Huang chickens, three chickens with high cloacal skin yellowness values were chosen and defined as the high yellowness (s_deep) group; another three chickens with low cloacal skin yellowness values were chosen and defined as the low yellowness (s_light) group. There was a significant difference in the average yellowness of the s_deep and s_light groups (12.53 ± 0.69 vs. 6.99 ± 0.66, *p* < 0.001, *n* = 3). A piece of skin (approximately 8 mm in diameter) was isolated from the cloaca, and the subcutaneous fat was scraped off and then snap-frozen in liquid nitrogen before RNA-seq was conducted. Oligo(dT)-attached magnetic beads were used to purify mRNA. The purified mRNA was fragmented into small pieces with fragmentation buffer at the appropriate temperature. Then, first-strand cDNA synthesis was performed. Thereafter, A-Tailing Mix and RNA-Index Adapters were added via incubation for end repair. The cDNA fragments obtained from the previous step were amplified by PCR, and the products were homogenized with Ampure XP Beads and then dissolved in EB solution. The products were validated in an Agilent Technologies 2100 system for quality control. The double-stranded PCR products from the previous step were heated, denatured and circularized via the splint oligo sequence to obtain the final library. The single-stranded circular DNA was formatted as the final library. The final library was amplified with phi29 to generate DNA nanoballs (DNBs), which included more than 300 copies of a single molecule. The DNBs were loaded into the patterned nanoarray, and single-end 50-base reads were generated on the BGI-500 platform (BGI-Shenzhen, China). Adaptors, reads with unknown base(N) > 5%, and low-quality sequences were removed using the FASTX tool, which is used to reduce data noise and to clean raw reads. Furthermore, SOPA2(version 2.2.1) was used to filter out potential rRNA reads. After filtering, Clean reads of the skin tissues of each color were aligned to the reference (GCF_000002315.4_Gallus_gallus-5.0) by TopHat (version 2.2.1). Gene expression was measured in fragments per kilobase of exon per million reads mapped (FPKM). Functional annotation of the assembled reference sequences was performed by homology searches against the NCBI Nr (Non-redundant protein) database, the UniProt-Swiss-Prot (The Universal Protein Resource) database, and the KEGG (Kyoto Encyclopedia of Genes and Genomes database). Searches were conducted by the BLAST program with an *E*-value cut-off of 1e^–5^. The gene name and description of the best blast hit were assigned to each contig with significant hits. Finally, edger22 was used to normalize the expression level of each gene in six skin samples to identify the differentially expressed genes by pairwise comparisons. The threshold values of | logFC| ≥1 and FDR (False Discovery Rate) ≤0.05 were used to judge the significance of DEGs. GO term enrichment and KEGG pathway analysis of DEGs was performed using KOBAS program. All original RNA-seq data were deposited in the NCBI Gene Expression Omnibus (GSE144982). Genes exhibiting a fold change ≥2 and adjusted *p* ≤ 0.001 were considered differentially expressed genes (DEGs). GO term enrichment and KEGG pathway analysis of the DEGs were performed using the online-accessible Dr. TOM program^1^.

### cDNA Synthesis and Quantitative Real-Time PCR

The reverse transcription reaction for mRNA was performed with the PrimeScript RT reagent Kit with gDNA Eraser (Takara) according to the manufacturer’s manual. All primers (Table 1) were designed using Primer Premier 5.0 software (Premier Biosoft, Palo Alto, CA, United States). The qPCR program was carried out in a Bio-Rad CFX96 Real-Time Detection System (Bio-Rad, Hercules, CA, United States) with iTaq^TM^ Universal SYBRGreen^®^ Supermix (Bio-Rad). The 2^–ΔΔCt^ method was used to measure gene expression with β*-actin* as the reference gene.

**TABLE 1:** Sequence of PCR primers used in this study.

**Gene**	**Forward primer sequence (5′−>3′)**	**Reverse primer sequence (5′−>3′)**	**Length (bp)**
*ACACB*	TTCGGGACTTCAACCGTGAG	GGCTGCTTAAAATCCCGCAG	142
*TLR2B*	GAAACTTTGAGGGCATTG	TTGCGAAAGAGGAAGACATA	252
*FHL2*	CCTGCTTTATCTGCTACCGCTG	CACACCTCCTGTAGTGATAGCCTT	149
*CYP11A1*	TCCGCTTTGCCTTGGAGTCTGTG	ATGAGGGTGACGGCGTCGATGAA	112
*SMOC1*	CAGCACTTGCCCATTCC	CACCTTCCGCAGTAACAC	174
β*-actin*	ATCTTTCTTGGGTATGGAGTC	GCCAGGGTACATTGTGG	133
*GPR143*	TCAACAGTGGGCGACAATGA	GCAGAGACATACGCCTGCTA	443
*TECRL*	GACACAAACCCTGCCAGTTG	GACCCACTTCGGTTGCTGAA	226
*TYR*	CGAGACACACTCTTAGGTGG	CTTCTGTATCTCACGTTCCC	120
*PMEL*	GCACGCAGTTCAGCATCACC	CCCGAAGTCCCACGAATAGG	179
*ALDH1A3*	AAAGGCAGGTGCTCCTAAGC	CGTTGCAAAGTGCTGACACA	109
*IYD*	TAACTGCCTCACCCTA	CCCATCCCTACATACC	*258*
*BCO2*	TCACGCTTTGATCCACCGAA	CGTTTCTGGGTCCACCTTGT	117

### Statistical Analysis

Descriptive statistical analysis was performed using all available yellowness values records by the SPSS 21.0 (SPSS Inc., Chicago, IL, United States). Pearson correlation coefficients (r) and linear regression were used to evaluate the correlation among traits by the SPSS 21.0. A *p*-value < 0.05 was considered statistically significant. For statistical analysis of two contrast, we using one-sample *t*-test through SPSS. We considered *p* < 0.05 to be statistically significant. ^∗^*p* < 0.05; ^∗∗^*p* < 0.01.

## Results

### Characteristic of Skin Yellowness in Ma-Huang Chicken

**FIGURE 1:**
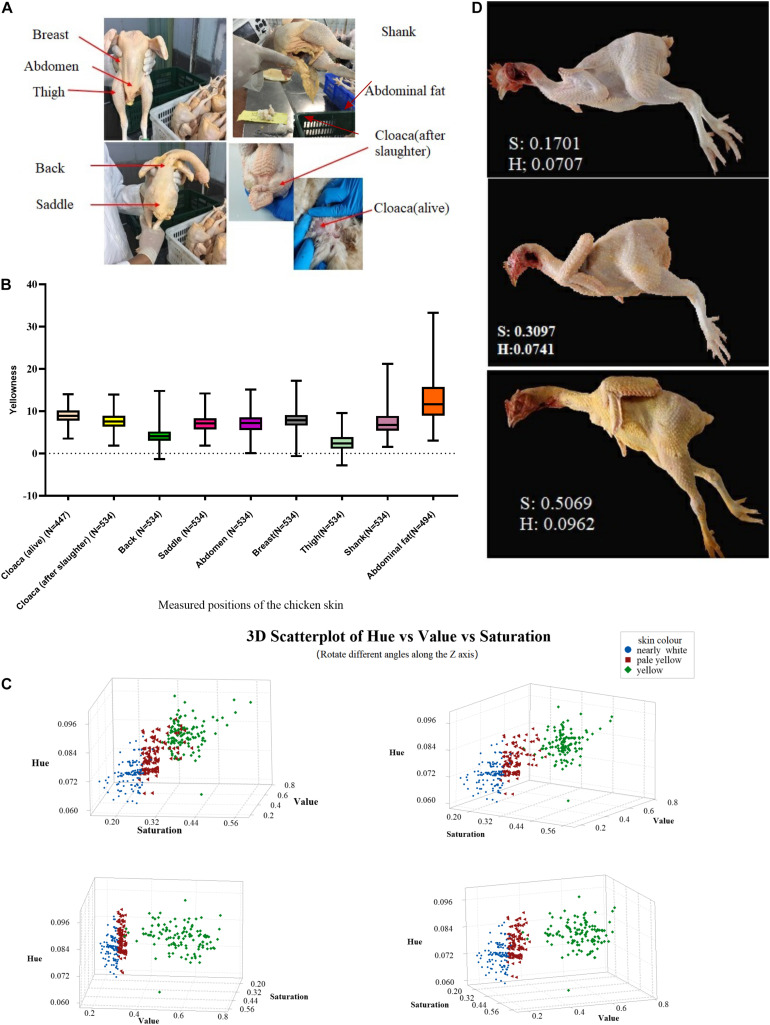
Characteristics of skin yellowness in Ma-Huang chickens. **(A)** schematic diagram of the positions at which skin yellowness was determined. **(B)** the b* value distribution for different body parts. **(C)** 3D Scatterplot of Hue vs saturation vs Value of skin color in Ma-Huang chickens (Rotate different angles along the Z axis). All samples were superimposed on the color space according to the hue, value and saturation values derived from the skin area assessed in digital photographs. According to the HSV ranking, the individual’s visual perception is divided into 3 parts, followed by nearly white (blue dots), pale yellow (red dots), and yellow (green dots). *n* = 360. **(D)** examples of skin photographs corresponding to the hue-saturation color space.

To understand the characteristics of skin pigmentation in chickens, we collected skin yellowness data from 534 Ma-Huang chickens. A total of eight different body skin positions were evaluated with a colorimeter (Figure 1A), and the discrete distribution of the yellowness value of each position is shown in Table 2. The results showed that the skin yellowness values varied among individuals (Figure 1B). Thigh skin exhibited the lowest mean yellowness values among the measured positions, whereas abdominal fat exhibited the highest mean yellowness values. Next, we used hue and saturation values that were collected from photographs and analyzed with MATLAB software to assess the overall skin color of individual chickens. To better classify the skin yellowness of the chickens, the depth of the skin color of Ma-Huang chickens was graded into three levels according to the HSV values as follows: “nearly white,” “pale yellow,” and “yellow” (Figure 1C) (according to the HSV ranking, the individual’s visual perception is divided into three parts. The specific skin color classification data are in the Supplementary Table 1). Within a subsample, there were 120 chickens with “nearly white” skin, 120 chickens with “pale yellow” skin, and 120 chickens with “yellow” skin. The overall hue value of the skin ranged from 0.06 to 0.10, and the overall saturation value of the skin ranged from 0.15 (very light) to 0.58 (yellow). To better understand the association between Hue-Saturation values and skin color, three representative chicken images were mapped to the 3D scatterplot space (Figure 1D). The darkness variation in saturation was easy to perceive, but there was little yellow-red variation in hue (Figure 1D). These data indicate that under intensive farming conditions, the saturation of the skin color of broilers presented a greater difference than the hue.

**TABLE 2:** Descriptive statistics of chicken skin color.

	**Cloaca (A)**	**Cloaca (as)**	**Back**	**Saddle**	**Ab**	**Breast**	**Thigh**	**Shank**	**AF**	**Hue**	**Saturation**
N	447	534	534	534	534	534	534	534	494	360	360
Minimum	3.51	1.88	−1.38	1.86	0.10	−0.64	−2.84	1.52	3.04	0.06	0.15
median	8.90	7.54	4.08	7.10	7.15	7.86	2.37	6.75	11.60	0.08	0.29
Maximum	13.98	13.90	14.78	14.19	15.09	17.21	9.56	21.21	33.24	0.10	0.58
mean	8.96	7.62	4.24	7.11	7.03	7.77	2.57	7.27	12.80	0.08	0.30
SEM	0.08	0.09	0.09	0.09	0.09	0.09	0.10	0.12	0.25	0.00	0.00
Range	10.47	12.02	16.16	12.33	14.99	17.85	12.40	19.69	30.20	0.04	0.43
Skewness	−0.06	0.29	1.11	0.42	0.02	−0.19	0.48	1.05	1.01	0.15	0.64
Kurtosis	−0.03	0.24	3.42	0.35	0.30	1.67	0.17	1.90	0.94	−0.25	0.66

*Cloaca (A), Cloaca (alive); Cloaca (as), Cloaca (after slaughter); Ab, Abdomen; AF, abdomen fat.*

### The Correlation Analysis of Skin Yellowness Between Different Parts of Chicken Skin

**FIGURE 2:**
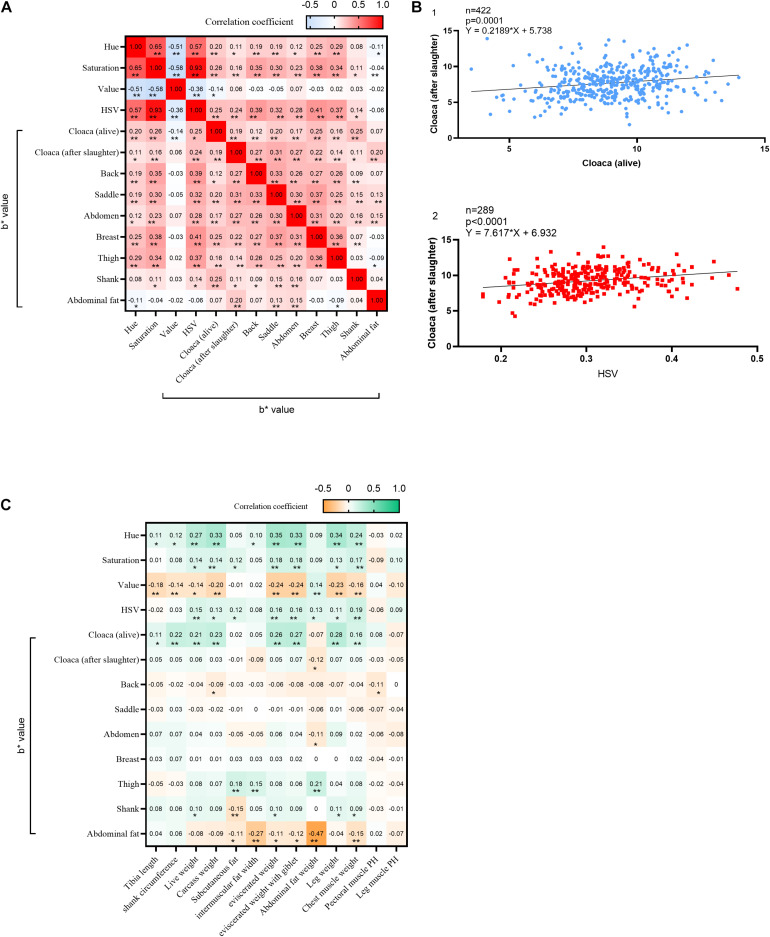
Correlation analysis of skin yellowness between different areas of chicken skin. **(A)** Pearson’s correlation coefficient (PCC) between the b* value of skin and hue and saturation. **p < 0.01, *p < 0.05; **(B)** analysis of the linear relationship between the cloaca before slaughter and the cloaca after slaughter, the yellowness of the cloaca after slaughter and HSV, and the yellowness of abdominal fat and the weight of abdominal fat; **(C)** Pearson’s correlation coefficient (PCC) between economic traits and skin color values, **p < 0.01, *p < 0.05.

To better understand the correlation of yellowness values among different positions and the correlation between skin yellowness and hue-saturation values, we conducted correlation analysis for each value. In general, there was a significant positive correlation among the yellowness values at each measured position, except for the V value and abdominal fat values (Figure 2A). Notably, the yellowness value of the cloaca before slaughter was positively correlated with the yellowness value of cloacal skin after slaughter as well as the yellowness values at other skin positions (Figure 2B). These results suggested that the yellowness value of the cloacal skin in a live chicken can serve as a reference for the overall skin yellowness value of the carcass. Then, we analyzed the correlation between skin color values and other economic traits of chickens. The yellowness value of the cloaca before slaughter was positively correlated with many traits, especially growth traits such as live weight and tibia length (Figure 2C). The HSV values were also positively correlated with these economic traits. However, we noted that many traits related to fat were uncorrelated with skin color traits, and the yellowness value of abdominal fat was significantly negatively correlated with abdominal fat weight. In summary, these results indicate that the skin yellowness values at different positions on the chicken are correlated with each other and that the abdominal fat weight is negatively correlated with skin color.

### Estimation of Genetic Parameters of Skin Yellowness Value

Considering that skin yellowness has a crucial effect on chicken sales, we evaluated the genetic parameters associated with the skin yellowness value. The heritability of the skin yellowness value was low, ranging from 0.07 to 0.27 (Table 3). Shank skin yellowness presented the lowest heritability, whereas abdominal fat yellowness presented the highest heritability (Table 3). Among all carcass traits, the carcass weight exhibited the highest heritability, and the heritability of the eviscerated weight (EW), eviscerated weight with giblets (EWG), and abdominal fat weight (AFW) ranged from 0.29 to 0.39 (Table 3).

**TABLE 3:** Estimates of heritabilities for the traits.

**Traits**	**Number**	***h*^2^**
Hue	349	0.13728 ± 0.09468
Saturation	349	0.24816 ± 0.11162
Cloaca (A) Yellowness	447	0.15422 ± 0.1027
Cloaca (as) Yellowness	534	0.11238 ± 0.06528
Saddle Yellowness	534	0.20576 ± 0.11324
Breast Yellowness	534	0.15762 ± 0.11784
AF Yellowness	534	0.2769 ± 0.12251
Shank Yellowness	534	0.07842 ± 0.01246
Back Yellowness	534	0.10862 ± 0.08554
Thigh Yellowness	534	0.11375 ± 0.04829
CW(kg)	534	0.51487 ± 0.13756
IFW(mm)	511	0.10165 ± 0.07356
EW(g)	510	0.2948 ± 0.10974
EWG(g)	510	0.38747 ± 0.11795
AFW(g)	510	0.35412 ± 0.10983
LMW(g)	510	0.17249 ± 0.09178
BMW(g)	510	0.1919 ± 0.09108

*Standard errors of estimates are in parentheses. Cloaca (A), Cloaca (alive); Cloaca (as), Cloaca (after slaughter); Ab, Abdomen; AF, abdomen fat; CW, carcass weight; IFW, intermuscular fat width; EW, eviscerated weight; EWG, eviscerated weight with giblet; AFW, abdominal fat weight; LMW, leg muscle weight; BMW, breast muscle weight.*

### Differentiated Expressed DEG Between Low Yellowness Skin and High Yellowness Skin

**FIGURE 3:**
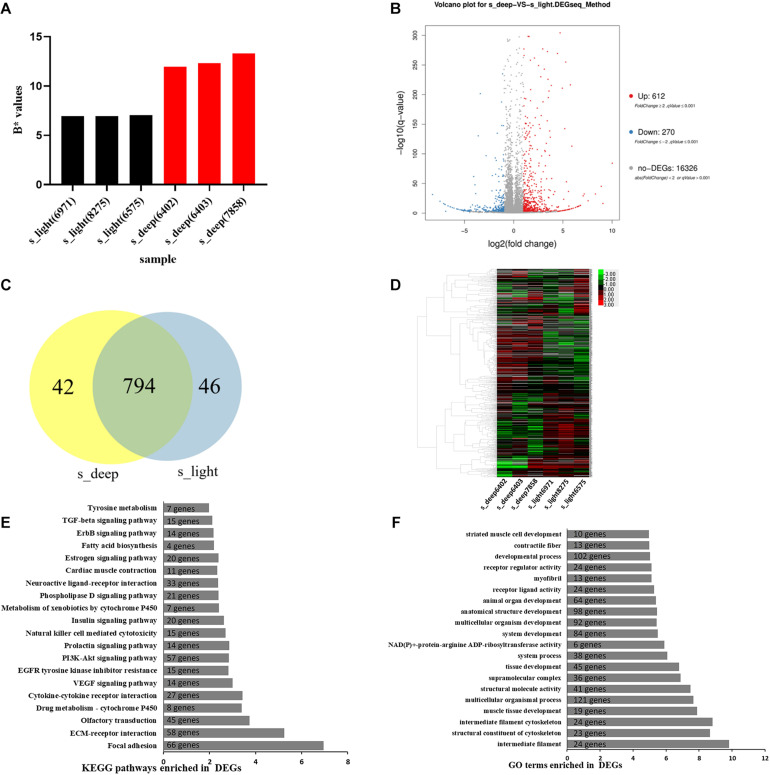
Differentially expressed genes between skin with low yellowness and high yellowness. **(A)** The b* value of RNA-seq samples. **(B)** volcano plot showing the DEGs between s_deep and s_light. The *X*-axis represents the difference multiplied after log2 conversion, and the *Y*-axis represents the significant values after log 10 conversion. Red represents upregulated DEGs, blue represents downregulated DEGs, and gray represents non-DEGs. **(C)** venn diagram representing DEGs overlapping between the two groups. **(D)** A heat map clusters the FPKM values of the differential genes in each comparison group. The horizontal axis represents the log2 (FPKM + 1) of the sample, and the vertical axis represents genes. The redder the color of the color block, the higher the expression, and the bluer the color, the lower the expression. **(E)** KEGG enrichment analysis of the DEGs between s_deep and s_light. The *y* axis represents KEGG pathways and the *x* axis represents | log(*p* value)|. The number of genes enriched in the indicated pathway was marked. **(F)** GO enrichment analysis of DEGs between s_deep and s_light. The *y* axis represents GO terms and the *x* axis represents | log(*p* value)|. The number of genes enriched in the indicated GO terms was marked.

To explore the genetic mechanism underlying the regulation of pigmentation, six RNA-seq libraries were obtained from the skin of chickens with high yellowness (s_deep) and low yellowness (s_light) (Figure 3A). There was a significant difference in the average yellowness of the s_deep and s_light groups (12.53 ± 0.69 vs. 6.99 ± 0.66, *p* < 0.001). During filtering, linker contamination, unknown base N content greater than 10%, and low-quality reads are sequentially removed (we define reads with a quality value of fewer than 15 bases that account for more than 50% of the total bases in the reads as low-quality reads). A total of 23.03 and 23.01 M valid reads were obtained from s_light and s_deep, respectively (Supplementary Table 2). According to a fold change ≥2 and an adjusted *p* ≤ 0.001, 882 unigenes were identified as differentially expressed between s_deep and s_light (Wang et al., 2010; Figure 3B). The 20 most significantly differentially expressed genes, including 10 upregulated and 10 downregulated, are listed in Table 4. Compared with the s_deep group, 612 genes were upregulated, and 270 genes were downregulated in the s_light group (Figure 3B) (fold change ≥2, adjusted *p* ≤ 0.001). Through integrative analysis of these DEGs between the s_deep and s_light group, we found that 794 DEGs were commonly expressed in both samples, whereas 42 DEGs and 46 DEGs were specifically expressed in the s_deep chickens and the s_light chickens, respectively (Figure 3C and Supplementary Table 3). These group-specific DEGs may be important for resulting in the different performance of pigmentation between the two group chickens. According to the results of the analysis of differentially expressed genes, hierarchical clustering analysis (HCA) was performed on the combined DEGs using the heatmap function in R software. HCA demonstrated diverse expression profiles between s_deep skin tissues and s_light skin tissues (Figure 3D).

**TABLE 4:** Detailed information of the top 20 most differentially expressed genes.

**Gene ID**	**Other Gene ID**	**s_deep FPKM**	**s_light FPKM**	**log2(s_light/s_deep)**	**Q value**	**P value**	**Up/Down**
421222	*MALL*	0	1.94	7.56	<0.001	<0.001	up
428512	*GALR1*	0.02	2.56	7.16	<0.001	<0.001	up
417428	*CACNG1*	0	1.14	6.76	<0.001	<0.001	up
430657	*KRTAP19-2*	0	1.59	6.43	<0.001	<0.001	up
423298	*ACTC1*	0.24	13.14	5.76	<0.001	<0.001	up
396472	*MYL4*	0.56	25.45	5.65	<0.001	<0.001	up
418726	*FHL2*	3.08	138.58	5.48	<0.001	<0.001	up
408032	*BVES*	0.34	13.20	5.29	<0.001	<0.001	up
100859398	*POLD2*	0.20	7.41	5.19	<0.001	<0.001	up
395814	*FMOD*	0.35	12.19	5.11	<0.001	<0.001	up
428125	*ART7C*	2.17	0.13	−4.06	<0.001	<0.001	down
396383	*ISL1*	1.54	0.13	−3.94	<0.001	<0.001	down
423899	*NRAP*	2.32	0.19	−3.6	<0.001	<0.001	down
693257	*GNLY*	25.37	2.39	−3.39	<0.001	<0.001	down
425968	*KRTAP10-4*	1.53	0.36	−2.09	<0.001	<0.001	down
424018	*PRLH*	2.26	0.53	−2.09	<0.001	<0.001	down
420894	*SERPINB1*	1.09	0.28	−1.96	<0.001	<0.001	down
107051294	*QPRT*	2.41	0.64	−1.89	<0.001	<0.001	down
395108	*GZMA*	2.29	0.65	−1.82	<0.001	<0.001	down
769322	*STARD4*	16.65	4.73	−1.81	<0.001	<0.001	down

To define the biological functions of the 882 DEGs, GO and KEGG enrichment analysis was performed with the phyper function of R software. Top 20 most significant pathways and GO terms were listed (Figures 3E,F). The KEGG pathway enrichment analysis showed that the DEGs were significantly involved in Focal adhesion, ECM-receptor interaction, olfactory transduction, drug metabolism-cytochrome P450, and cytokine-cytokine receptor interaction (Figure 3E). Notably, there are two enriched pathways related to cytochrome P450 in this result (Figure 3E). Previous studies have shown that the gene encoding the cytochrome P450 enzyme was found to associate with carotenoid-based avian coloration phenotypes (Lopes et al., 2016; Mundy et al., 2016). Therefore, these cytochrome P450 related pathways may play an important role in the regulation of the chicken yellow skin trait. GO analysis showed that intermediate filament, structural constituent of cytoskeleton, intermediate filament cytoskeleton, muscle tissue development, and multicellular organismal process were enriched in the functions of DEGs (Figure 3F). Furthermore, *tyrosinase* (*TYR*), *pre-melanosomal protein* (*PMEL*), and *G protein-coupled receptor 143* (*GPR143*), which are involved in the regulation of pigmentation, were significantly differentially expressed between the two groups (Supplementary Table 3).

### Gene Interaction Networks Involved in the Regulation of Skin Yellowness

**FIGURE 4:**
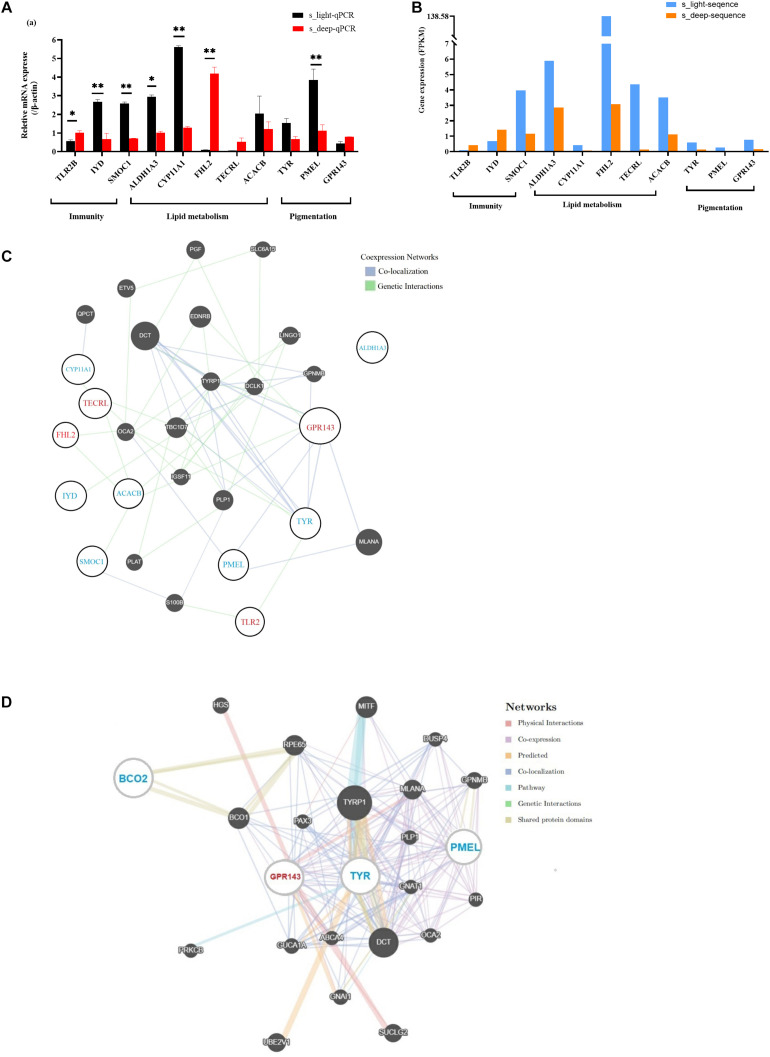
DEGs and pathways involved in the regulation of chicken skin yellowness or carotenoid pigmentation. **(A)** quantification of the mRNA levels from the RNA extracted from the s_light and s_deep samples. **(B)** gene expression patterns obtained using RNA-Seq. **(C)** interaction network of the DEGs involved in the regulation of immunity, carotenoid metabolism, and melanin metabolism. **(D)** interaction network of DEGs involved in pigmentation. Compared with the s_light group, the expression of genes marked in red increased in the s_deep group, while blue decreased. Genes that did not belong to the DEG list are potential target genes indicated in white font. * 0.01 < *p* < 0.05, **p < 0.01.

Studies have shown that immunity and lipid metabolism are related to skin pigmentation. To validate our RNA-seq results and find some candidate genes involved in skin color regulation, we selected 11 DEGs implicated in immunity, lipid metabolism, and pigmentation for qPCR confirmation. We observed consistency between the qPCR results and RNA-seq results (Figures 4A,B). Next, by using GeneMANIA, we generated a protein interaction network to show how these eleven DEGs communicated or interacted with each other (Figure 4C). This network contributes to our understanding of the regulatory mechanism linking pigment deposition and immunity in chickens. Additionally, we also used GeneMANIA software to predict the potential target genes of *TYR*, *PMEL*, *GPR143*, and *BCO2*, which were involved in pigmentation. Then we generated an interaction network consisting of these four genes and their potential targets (Figure 4D). This network may play roles on chicken skin carotenoid deposition. Within this network, *TYR*, *PMEL*, and *GPR143* were differentially expressed between s_light and s_deep according to the RNA-sequencing results (Supplementary Table 3). *PMEL* and *GPR143* are mainly involved in the regulation of melanin deposition, and their roles in carotenoid deposition have rarely been reported. Therefore, based on our interaction network, it will be an important research content to study how *PMEL* and *GPR143* interact with *TYR* and *BCO2*, and ultimately affect the carotenoid deposition in chicken skin. The differential expression of these genes may lead to an imbalance in the network and ultimately affect yellow pigment deposition.

## The Expression and the Three Typical SNPs of BCO2 Cannot Use to Estimate the Chicken Skin Yellowness in This Chicken Population

**FIGURE 5:**
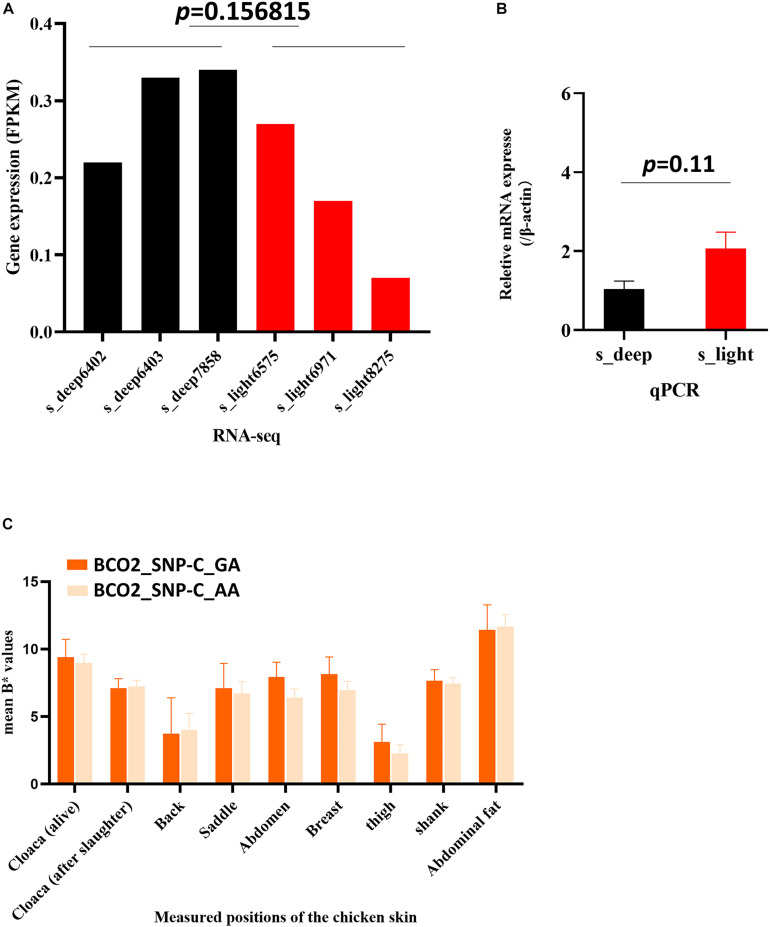
The expression and variation of *BCO2* cannot be used to estimate chicken skin yellowness in this chicken population. **(A)** RNA-seq sequencing results showed that the expression of *BCO2* is no significant difference between s_deep and s_light samples. *p* value = 0.156815, one-sample *t* test was used to assess the difference between the s_deep and s_light samples. **(B)** qPCR results of the *BCO2* gene (*p* = 0.11) *n* = 3. One-sample *t* test was used to assess the difference between the s_deep and s_light samples. **(C)** Skin yellowness value (B* value) in chickens with GA genotype and AA genotype. The skin yellowness has no significant difference between chickens with GA genotype and AA genotype.

*BCO2* plays an important role in determining the yellow skin of chickens. Mutations in the region of the *BCO2* gene can result in a phenotypic change. Eriksson et al. (2008) reported that three single-nucleotide polymorphisms (SNPs: A, B, and C) around the *BCO2 g*ene were associated with the yellow skin phenotype and that low *BCO2* expression allows the deposition of yellow carotenoids in the skin. However, our RNA-seq and qPCR results showed that the *BCO2* yellow skin gene was not significantly differentially expressed between the s_deep samples and the s_light samples (Figures 5A,B). To further test whether the three *BCO2* SNPs are correlated with the skin yellowness value in this experimental group, we selected 40 two-tailed samples of skin yellowness values for verification (20 chickens with highest yellowness values and 20 chickens with lowest yellowness values). The genotyping results showed that SNP-A and SNP-B were the same in all 40 chickens, and only SNP-C showed polymorphism in these 40 chickens (Supplementary Table 4). Next, we compared the skin yellowness value between the chickens with GA genotype and the chickens with AA genotype. The result showed that there is no significant difference in skin yellowness value between chickens with different type of SNP-C (Figure 5C). Correlation analysis results also showed that the SNP-C was not correlated with skin yellowness value in any area (Supplementary Table 5). Therefore, these results suggest that the expression and the three typical yellow skin SNPs of *BCO2* may not be used to estimate chicken skin yellowness.

## Discussion

Skin color makes phenotypic selection difficult in chickens because the skin of chickens is covered with feathers. Several positions covered with few feathers can be used to measure the skin color directly, such as the skin around the cloaca and the skin of the underwing. However, there is a vein under the skin of the underwing, which can significantly affect the color value detection. Therefore, the skin around the cloaca may be the only position at which skin color can be directly measured in a live chicken. Importantly, there are no blood vessels under the skin around the cloaca, and our correlation analysis results showed that the skin yellowness value at this position was correlated with overall chicken skin color after slaughter. These results suggest that the cloacal skin yellowness of a live chicken may be a useful value for assessing the overall skin yellowness of a slaughtered chicken, and may imply for application in deep yellow skin chicken selection.

At present, research on the skin color of poultry mainly depends on the use of a colorimeter and color plate to assess skin color after slaughter. For example, Sirri et al. (2010) used a CR-300 chromameter to determine the skin color of the breast, thighs, and shanks. Mueller et al. (2020) used a CR-300 chromameter to detect the skin color of the left breast to evaluate the skin color differences among different broiler breeds. The skin color detection position employed in the experiment of Rajput et al. was the same as employed by Sirri et al. (Rajput et al., 2013). However, a colorimeter can only determine the color within a small piece of skin, and the color plate measurement is influenced by subjective factors. To overcome these defects, we developed a method combining high-resolution photos and MATLAB software valuation to quantify the global skin color of chickens. A similar method has been employed in human skin color research (Jacobs et al., 2013). On the basis of this method, we can use a single number to represent the skin color of a chicken. In addition, we found that many skin yellowness values determined by the colorimeter were positively correlated with the global skin color value analyzed by MATLAB, suggesting that the skin yellowness value of a single position is representative to some extent. Additionally, our results showed that the skin yellowness values detected at different positions were positively correlated with each other. Díaz-Gómez et al. (2017) also found that there was a correlation between breast skin yellowness and thigh skin yellowness in chickens. Therefore, Pigments may evenly deposit in different areas of the chicken body skin.

Broilers are prone to excessive fat deposition, but most of this fat is not physiologically necessary. Yellow fat is occasionally observed in cattle, sheep, pigs, and rabbits, but it occurs widely in poultry (Strychalski et al., 2016). Broilers deposit a portion of the adsorbed dietary pigments into the fat. Female broilers exhibit higher subcutaneous fat levels than males (Fletcher, 1992). Our experiment showed that the yellowness value of abdominal fat presents a very significant negative correlation with abdominal fat weight (Figure 2), which means that more abdominal fat deposition will lead to lighter skin color. The potential underlying mechanism may be the dilution of the pigment deposited in the fat by lipids, resulting in a decrease in the yellowness value.

The *BCO2* gene encodes beta-carotene dioxygenase 2, which cleaves carotenoids (colorful substance) to apocarotenoids (colorless substance) via an asymmetrical cleavage reaction (Kiefer et al., 2001), is detected in many tissues and can affect color trait (Lindqvist et al., 2005). Kong et al. (2017) reported that the frequency of the reported SNPs (SNP A, SNP B, SNP C) of BCO2 was significantly different between yellow- and white-skinned. Using CRISPR/Cas9 to knock out the *EcBCO2* gene can lead to a color change in the hepatopancreas of prawns (Sun et al., 2020). However, there are also studies showing that *BCO2* is not related to yellow skin traits. For example, Yang et al. (2012) found that one SNP of *BCO2* (chr24: 6273428) was the same among three yellow skin chicken breeds. Xu et al. (2015) reported that the three classic SNPs of *BCO2* and the yellow skin traits of Gushi chickens were not statistically correlated. In our experiment, both mRNA expression and association analysis showed that *BCO2* and skin yellowness values were not correlated. Kiefer et al. (2001) proposed that when the expression of *BCO2* is low in skin tissue, carotenoids remain intact and will be incorporated into keratinocytes. We speculate that *BCO2* may determine whether the skin is yellow or white but cannot determine the yellowness value of skin. The skin yellowness of chickens may be a quantitative trait regulated by multiple genes.

Here, we found that the yellowness value of cloaca skin in live chicken is significantly correlated with skin yellowness of chicken after slaughter. However, this result still need further verified. We are planning to use the yellowness value of cloaca skin in live chicken to select chickens and divide them into high and low yellowness groups. The chickens will then be slaughtered and detected for the yellowness value of their skin. If the high yellowness group has significantly higher yellowness value than those in low yellowness group after slaughtered, this method would be implied for application in yellow skin chicken selection. The lighter the skin color, the cheaper the chicken is sold in South China. Chicken breeding companies can weed out chickens with low yellowness value, therefore, resulting in more uniform skin color in the flock.

In addition, considering the low heritability of skin yellowness that our results showed, it would take much time and require much more records to improve this trait in chicken breeding. Phenotypes such as skin yellowness value, carotenoid levels in the blood, health condition, and composition of pigment additives in feed can be recorded and use to guide the breeding selection. Besides, molecular markers can also be used in chicken yellow skin breeding. Even though the three SNPs of *BCO2* were not correlated with chicken skin yellowness in this study, other SNPs may be useful. DEGs found in our results, especially those genes included in the network, can be potential candidates to scan SNP and do the association analysis with chicken skin yellowness.

## Data Availability Statement

The datasets presented in this study can be found in online repositories. The names of the repository/repositories and accession number(s) can be found below: https://www.ncbi.nlm.nih.gov/genbank/, GSE144982.

## Ethics Statement

The animal study was reviewed and approved by the Animal Care Committee of South China Agricultural University (approval number: SCAU#0106; 25 November 2018).

## Author Contributions

JW and WL conceived, designed, and wrote the manuscript. JW, ZL, and GC performed the experiments. GC, QL, and ZL participated in the critical revising. QL, QN, XZ, and WL participated in revising and coordination. All authors reviewed and approved the final version.

## Conflict of Interest

The authors declare that the research was conducted in the absence of any commercial or financial relationships that could be construed as a potential conflict of interest.

## Footnotes

1 http://report.bgi.com
