# Supplementary figures and images for: Characterization of Chicken Skin Yellowness and Exploration of Genes Involved in Skin Yellowness Deposition in Chicken
Source: Front Physiol. 2021 Mar 31;12:585089. doi: 10.3389/fphys.2021.585089 (PMC8044320; doi:10.3389/fphys.2021.585089)

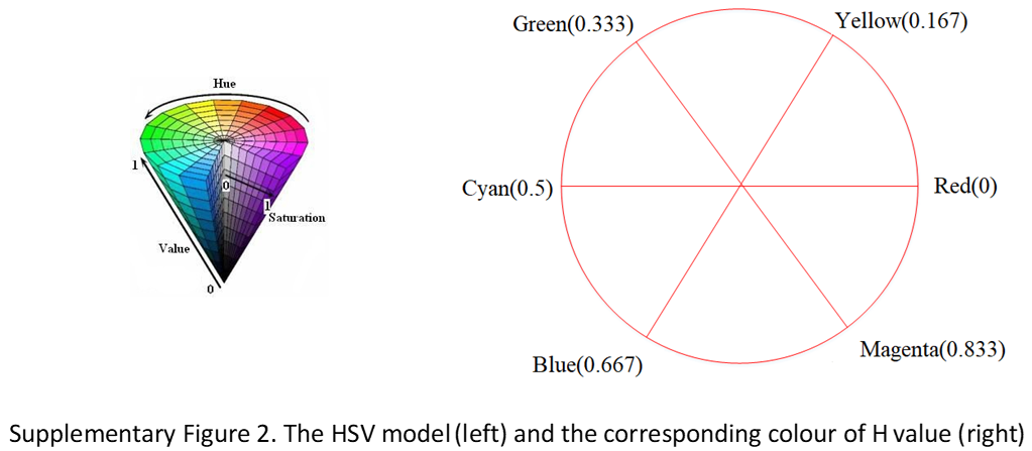

Supplement: Supplementary file 1 [file Image_1.PNG]

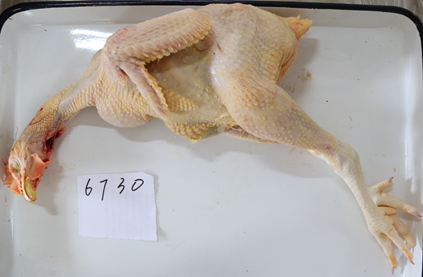

Supplement: Supplementary file 2 [file Image_2.PNG]
